# Development of a genetic transformation system in *Echinochloa phyllopogon* by particle bombardment

**DOI:** 10.1007/s00299-026-03978-4

**Published:** 2026-10-14

**Authors:** Takahiro Gondo, Manami Sugahara, Hwan May Ng, Kentaro Yasuda, Satoshi Iwakami

**Affiliations:** 1 Frontier Science Research Center, University of Miyazaki, 1-1 Gakuenkibanadai-Nishi, Miyazaki, 889-2192 Japan; 2 Faculty of Agriculture, University of Miyazaki, Miyazaki, Japan; 3 Faculty of Regional Innovation, University of Miyazaki, Miyazaki, Japan; 4 Agri-Innovation Education and Research Center, Akita Prefectural University, Akita, Japan; 5 Institute of Agriculture, Tokyo University of Agriculture and Technology, Tokyo, Japan; 6 United Graduate School of Agricultural Science, Tokyo University of Agriculture and Technology, 3-5-8 Saiwai-Cho, Fuchu, Tokyo 183-8509 Japan

**Keywords:** *Echinochloa phyllopogon*, Grass weed, Genetic transformation, Particle bombardment, Embryogenic callus, Herbicide resistance

## Abstract

**Key Message:**

A stable particle bombardment-mediated transformation system was developed for *Echinochloa phyllopogon*, providing a platform for functional analysis of genes involved in herbicide metabolic detoxification.

**Abstract:**

*Echinochloa phyllopogon* is a major C4 grass weed that causes serious yield losses in paddy fields and has evolved resistance to multiple herbicides. Understanding the molecular mechanisms underlying this resistance requires functional analysis of candidate genes involved in herbicide metabolic detoxification. However, the lack of a stable transformation system has limited such approaches. In this study, we established a particle bombardment-mediated transformation system for *E. phyllopogon* using embryogenic calli and plasmid pAHC25 carrying the *GUS* reporter gene and the bialaphos resistance gene (*bar*) selectable marker. Three morphologically distinct callus types were identified, among which Type 1 (compact) and Type 2 (friable) embryogenic calli exhibited significantly higher regeneration ability and transient GUS expression than Type 3 calli (wet and viscous). This finding indicates that callus quality is a critical determinant of transformation efficiency. In addition, callus culture duration significantly affected transformation outcomes. Calli cultured for 90 days produced the highest transformation efficiency (5.0%) and retained full regeneration capacity, whereas prolonged culture (300 and 630 days) resulted in reduced regeneration and transformation efficiency. Stable transgene integration and expression were confirmed by Southern blot analysis and histochemical GUS staining. Furthermore, transgenic plants exhibited strong resistance to Basta in the T_1_ generation, indicating that the herbicide-resistant phenotype was transmitted to the next generation. These results demonstrate that both callus type and culture duration are key factors for efficient transformation. The transformation system established in this study provides a valuable platform for functional analysis of genes involved in herbicide metabolic detoxification in *E. phyllopogon*.

## Introduction

Weed control is essential for maintaining agricultural productivity and meeting the increasing global demand for food. In modern agriculture, weed management relies heavily on herbicides, which have been widely adopted as efficient and cost-effective tools for weed control. However, the continuous and intensive use of herbicides has led to the widespread evolution of herbicide-resistant weeds, posing a serious challenge to sustainable crop production (Powles and Yu 2010).

Herbicide resistance mechanisms are broadly classified into target-site resistance (TSR) and non-target-site resistance (NTSR) (Yuan et al. 2007). TSR is caused by mutations in genes encoding herbicide target proteins, and most of the mutations have been successfully identified through sequence analysis of known or readily determined target genes (Baucom 2016). In contrast, NTSR encompasses a range of mechanisms, including reduced herbicide absorption and translocation, as well as enhanced detoxification capacity. Recent advances in high-throughput technologies, particularly transcriptomic analyses, have enabled comprehensive identification of genes associated with these processes, providing insights into mechanisms such as reduced herbicide movement, impaired foliar uptake, and metabolic detoxification (Gaines et al. 2020). However, the molecular basis of NTSR remains poorly understood, particularly with respect to the genes and regulatory mechanisms responsible for metabolic resistance (Rigon et al. 2020).

*Echinochloa phyllopogon* (Stapf.) Koss., (also known as *E. oryzicola*) is a self-pollinating allotetraploid C4 grass weed (2*n* = 4*x* = 36) commonly infests paddy fields (Yamasue 2001). It is one of the best-studied weed species for investigating the molecular mechanisms of metabolic herbicide resistance. Herbicide-resistant populations of *E*. *phyllopogon* were first reported in California in the late 1990s, and are recognized as one of the earliest cases of multiple herbicide resistance in weeds (Fischer et al. 2000a). These populations exhibit resistance to herbicides with diverse modes of action, including acetolactate synthase (ALS) inhibitors, acetyl-CoA carboxylase (ACCase) inhibitors, very-long-chain fatty acid (VLCFA) elongase inhibitors, 1-deoxy-D-xylulose 5-phosphate synthase (DXS) inhibitors, and synthetic auxins (Fischer et al. 2000b; Osuna et al. 2002; Ruiz-Santaella et al. 2006; Bakkali et al. 2007; Yasuor et al. 2009, 2010, 2012). This broad-spectrum resistance is largely attributed to enhanced herbicide metabolism, a form of NTSR that has also been increasingly reported in other weed species. Metabolic resistance poses a serious threat to sustainable agricultural production, as it can confer cross-resistance to multiple herbicides with different modes of action. Therefore, *E. phyllopogon* has emerged as an important model species for elucidating the molecular basis of metabolic herbicide resistance to develop effective weed management strategies (Dimaano and Iwakami 2021).

Recent genomic and transcriptomic studies have identified three cytochrome P450 genes, including *CYP81A12*, *CYP81A21*, and *CYP709C69*, as strong candidate genes involved in herbicide detoxification and metabolism in multiple herbicide-resistant populations of *E. phyllopogon*, particularly the California-derived line 511 (Iwakami et al. 2014, 2019; Guo et al. 2019; Dimaano et al. 2020; Suda et al. 2023; Chayapakdee et al. 2026). These studies further suggested that the coordinated overexpression of these P450 genes may be triggered by a single evolutionary event, such as a regulatory mutation, thereby contributing to the establishment of multiple herbicide resistance. However, direct functional validation of candidate genes in *E. phyllopogon* remains limited.

A major barrier to elucidating the molecular basis of NTSR in agricultural weeds is the lack of efficient genetic transformation systems. To date, functional studies in weed research have largely relied on model species such as rice and Arabidopsis (e.g., Han et al. 2021; Figueiredo et al. 2022; Suda et al. 2023). Although virus-mediated transient expression systems provide a rapid approach for preliminary functional analysis in several weed species (Mellado-Sánchez et al. 2020; Liu et al. 2026), stable transformation remains the most powerful approach for evaluating gene function and heritable phenotypes. Tissue culture and plant regeneration systems have been developed for several *Echinochloa* species, including *E. oryzicola* currently recognized as *E. phyllopogon* (Takahashi et al. 1984; Cobb et al. 1985), *E. muricata* (Cobb et al. 1985) and *E. glabrescens* (Wang and Zapata 1987), but a stable genetic transformation system has yet been established for the genus.

In this study, we established a stable and efficient genetic transformation system for *E*. *phyllopogon*, including reproducible callus induction, plant regeneration, and stable transgene expression. To our knowledge, this is the first report of a genetic transformation system in this species. This system provides a critical foundation for the functional genomic analyses involved in herbicide metabolic detoxification and will facilitate future studies aimed at identifying the molecular mechanisms underlying multiple herbicide resistance of NTSR in weeds.

## Material and methods

### Plant materials

The plant material used in this study was a herbicide-sensitive accession of *E. phyllopogon* (AEC02-17), collected from a paddy field in Nagano prefecture, Japan. Seeds were dehulled and surface-sterilized with 70% (v/v) ethanol for 30 s, followed by 1% (v/v) sodium hypochlorite for 15 min, and rinsed five times with sterile distilled water. Sterilized seeds were placed on filter paper moistened with 5 mL sterile distilled water and incubated at 31 °C under light conditions. Three days after germination, elongated roots and shoots were removed, and the basal tissues containing the shoot apical meristem were used as explants for callus induction.

### Callus induction and culture conditions

Callus induction was performed using Murashige and Skoog (MS) medium supplemented with 3% (w/v) sucrose, 0.3% (w/v) gellan gum, 2 mg L⁻^1^ 2,4-dichlorophenoxyacetic acid (2,4-D), 0.1 mg L⁻^1^ 6-benzylaminopurine (BAP), and 5 µM CuSO₄ (DBC medium). Explants were cultured on DBC medium at 30 °C in the dark. After 14 days of culture, elongated roots and shoots were removed, and the primary calli were transferred to fresh DBC medium. Subsequently, the resulting calli were classified into three types according to morphology: compact (Type 1), friable (Type 2), and wet and viscous (Type 3)—and subcultured at 14-day intervals.

### Plant regeneration from callus cultures

For plant regeneration, calli from each of the three callus types were transferred to MS medium supplemented with 0.02 mg L⁻^1^ naphthaleneacetic acid (NAA) and 2 mg L⁻^1^ kinetin (NK medium). Cultures were incubated at 27 °C under a 16-h light/8-h dark photoperiod. Each treatment consisted of three biological replicates, with 63 calli per replicate. After 28 days, the numbers of regenerated calli and shoots were recorded. Regenerated shoots were transferred to hormone-free half-strength MS (1/2 MS) medium for rooting and subsequently transplanted into 6-cm-diameter plastic pots filled with soil. Plants were acclimatized in a growth chamber at 27 °C under a 16 h light/8 h dark photoperiod.

### Bombardment with a particle inflow gun

Particle bombardment was performed using a particle inflow gun developed by Akashi et al. (2002). Approximately 25 calli were used per bombardment. Four hours prior to bombardment, calli were transferred to DBC medium supplemented with 0.3 M mannitol and 0.3 M sorbitol. Following bombardment, calli were maintained on the same medium for 16 h as an osmotic post-treatment.

Bombardment was carried out under the following conditions: helium pressure, 5.0 kg cm⁻^2^; target distance, 9.6 cm; chamber pressure, −0.1 MPa; one shot per dish. The plasmid pAHC25 (Christensen and Quail 1996), containing the β-glucuronidase (GUS) gene and the bialaphos resistance (*bar*) gene, each driven by the maize polyubiquitin-1 (*ubi-1*) promoter and its first intron, was used for transformation. Plasmid DNA was coated onto 1.0-µm gold particles (Bio-Rad) using ethanol precipitation method. The coated particles were suspended in 99% ethanol using sonication, and 20 µL of the suspension containing 0.8 µg plasmid DNA and 0.2 mg gold particles was used per shot (Gondo et al. 2005).

### Selection and regeneration of transgenic plants

Following bombardment and osmotic post-treatment, embryogenic calli were cultured on the DBC medium containing 5 mg L^−1^ bialaphos and subcultured at 14-days intervals. After 90 days of subculture under selective conditions, bialaphos-resistant callus clumps were transferred to NK medium supplemented with 5 mg L^−1^ bialaphos and cultured for approximately 30 days for shoot regeneration. All regenerated shoots were transferred to 1/2 MS medium for rooting. After 14–21 days, rooted plants were acclimatized in soil and maintained in a growth chamber at 27 °C under a 16-h light/8-h dark photoperiod.

### Histochemical GUS assay

Histochemical GUS assay was performed following a modified method of Jefferson (1987). To assess transient GUS activity, tissues were incubated for 18–24 h at 37 °C in a staining solution containing 1.9 mM 5-bromo-4-chloro-3-indolyl-β-D-glucuronic acid (X-Gluc), 50 mM phosphate-buffered saline (PBS; pH 7.0), 0.5 mM potassium ferricyanide, 0.5 mM potassium ferrocyanide, 0.3% (v/v) Triton X-100, and 20% (v/v) methanol, beginning 16 h after bombardment. To evaluate stable GUS expression, bialaphos-resistant calli and leaves of transgenic plant were stained using the same procedure.

### Southern blot analysis

Genomic DNA was extracted from leaf tissue using the CTAB method (Murray and Thompson 1980). Four micrograms of genomic DNA were digested with *SacI*, separated on a 0.8% agarose gel, and transferred onto Hybond nylon membranes (Roche, Germany). Membranes were hybridized with a 452 bp *GUS* gene fragment labeled using a PCR DIG Probe Synthesis Kit (Roche, Germany), amplified with primers 5′-GCCGGCCTACTGAAGGATC-3′ and 5′-TGCCTTCCTCATCCACGCA-3′. Hybridization signals were detected using the ChemiDoc Imaging System (Bio-Rad).

### Herbicide resistance assay in the T_1_ generation

Transgenic plants were grown in a growth chamber at 27 °C under a 16-h light/8-h dark photoperiod until sufficient vegetative growth had been achieved. Flowering was induced under a 12-h light/12-h dark photoperiod, and self-pollinated seeds were collected. Seeds from transgenic line #2 were used for the herbicide resistance assay, with wild-type (WT) plants serving as a control. Five seeds were sown in 12-cm pots with four replicate pots per treatment. Basta (BASF) was applied at the four-leaf stage at two concentrations: the recommended concentration (× 1; 1.02 kg a.i./ha) and at a ten times the recommended concentration (× 10; 10.2 kg a.i./ha). Plant responses were evaluated by photography 15 days after treatment.

## Results

### Embryogenic callus induction and callus classification

**Fig. 1:**
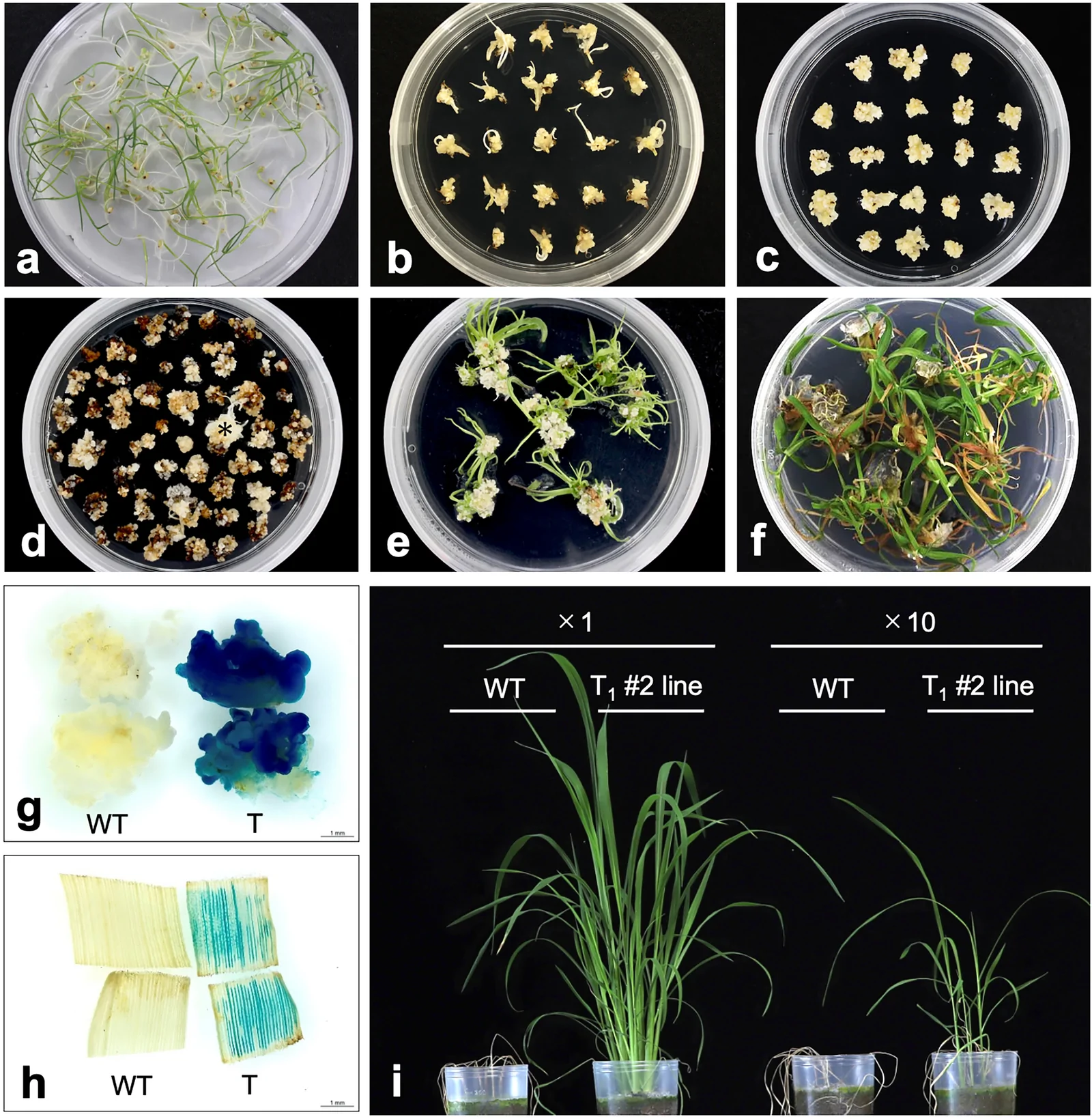
Stable genetic transformation of *Echinochloa phyllopogon*. **a** Germinated seedling after 3 days of culture on filter paper containing sterile water at 31 °C under the light conditions. **b**, **c** Primary calli after 14 days of culture (**b**) and embryogenic calli 28 days of culture (**c**) on MS medium containing 2 mg L^−1^ 2,4-D + 0.1 mg L^−1^ BAP + 5 µM CuSO_4_ (DBC medium) at 31 °C under dark conditions. **d** Bialaphos-resistant callus on DBC medium with 5.0 mg L^−1^ bialaphos. Bialaphos-resistant callus is indicated by asterisk (*). **e** Plant regeneration from bialaphos-resistant calli on MS medium containing 0.02 mg L^–1^ NAA, 2.0 mg L^−1^ Kinetin and 5.0 mg L^−1^ bialaphos. **f** Rooting of regenerated transgenic plants on half-strength MS medium. **g**, **h** Stable GUS expression on bialaphos-resistant callus (**g**) and leaves of transgenic plants (**h**) (WT, non-transgenic wild type line; T, transgenic line). **i** Herbicide resistance in the T_1_ generation of transgenic plants. WT and T_1_ #2 transgenic line after 15 days spraying with BSF basta (× 1, 1.02 kg a.i./ha; ×10, 10.2 kg a.i./ha)

**Fig. 2:**
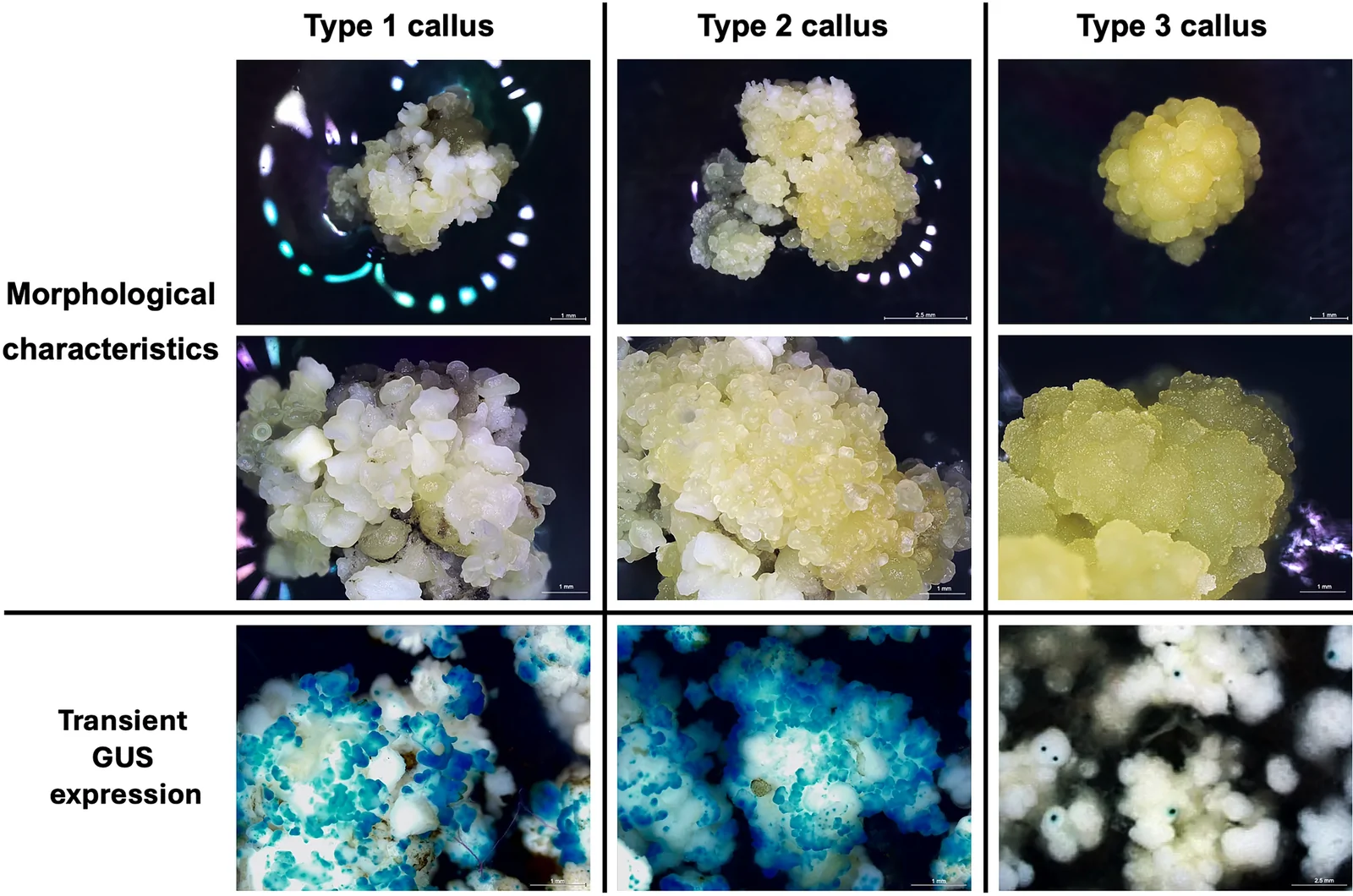
Morphological characteristics and transient GUS expression of different callus types in *Echinochloa phyllopogon*. Morphological characteristics of the three callus types were following. Type 1 callus was compact and consisted of clusters of somatic embryos. Type 2 callus was friable and highly proliferative, containing dense immature embryogenic clusters. Type 3 callus was wet and viscous. Transient GUS expression 16 h after particle bombardment in Type 1, Type 2, Type 3 calli. Type 1 and Type 2 calli showed higher levels of transient GUS expression, predominantly in regions containing dense immature embryogenic clusters

Three days after germination (Fig. 1a), seeds with the shoot apical meristem were cultured on DBC medium under dark conditions at 30 °C. After 14 days, primary calli were induced from tissues surrounding the meristematic region (Fig. 1b). After 28 days, embryogenic calli were formed (Fig. 1c) at a frequency of approximately 20–30% (data not shown). The induced calli were classified into three types based on their morphology (Fig. 2). Type 1 calli were compact and composed of clusters of somatic embryos. Type 2 calli were friable and highly proliferative, containing densely packed immature embryogenic tissues. In contrast, Type 3 calli were wet and viscous. All callus types were subcultured at 14-day intervals and used for subsequent experiments.

### Plant regeneration from different callus types

**Fig. 3:**
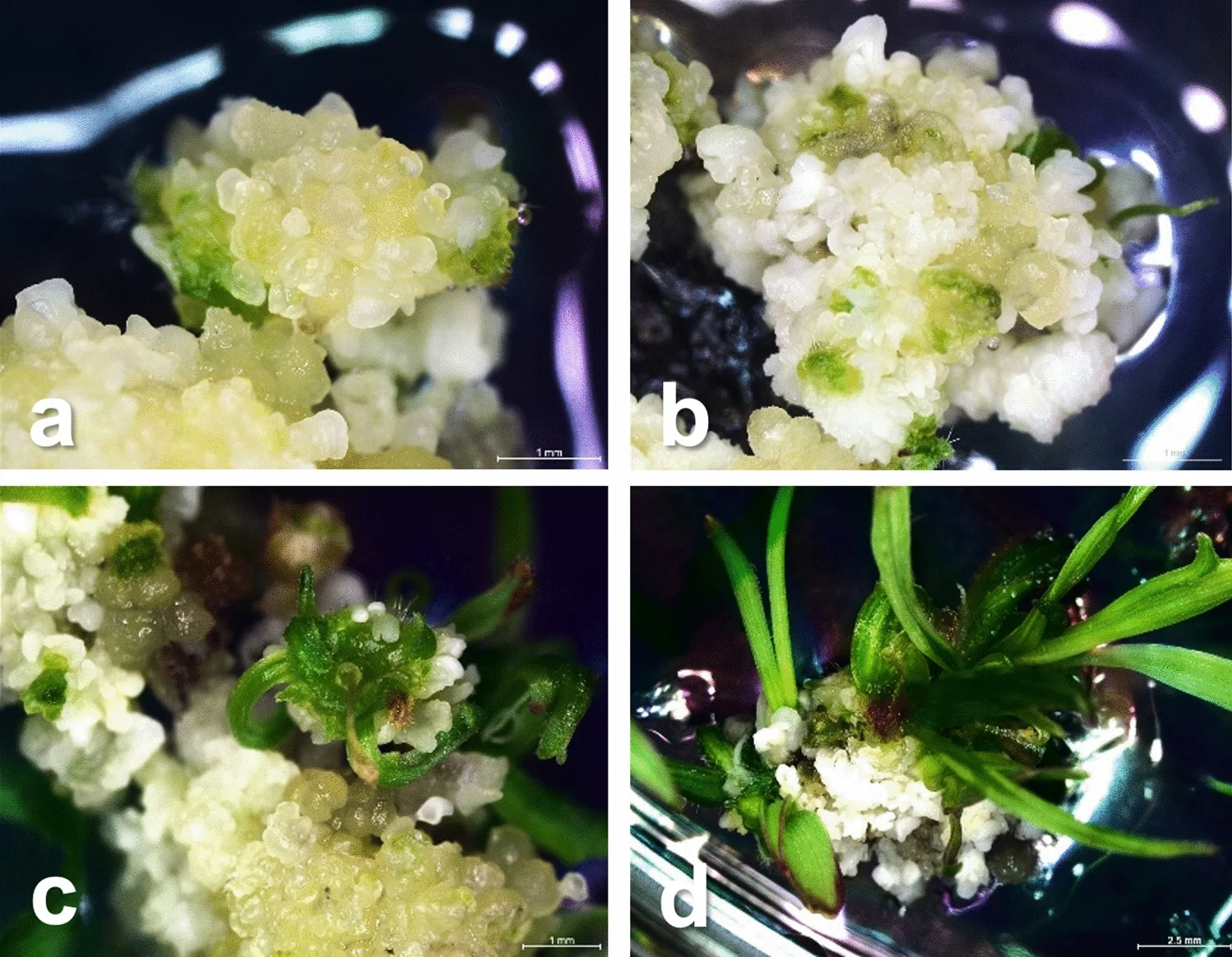
Developmental stages of plant regeneration from Type 2 calli for 0 (**a**), 7(**b**), 14 (**c**), 21 days (**d**) on MS medium containing 0.02 mg L^–1^ NAA and 2.0 mg L^–1^ Kinetin at 27 °C under light condition

Plant regeneration capacities of the three callus types were evaluated on NK medium. Type 1 and Type 2 calli showed high regeneration potential, producing approximately four shoots per callus, and exhibited significantly higher regeneration rates and shoot numbers than Type 3 calli (*p* < 0.05) (Table 1). The regeneration process in Type 2 calli is shown in Fig. 3. Immature somatic embryos, formed through callus induction (Fig. 3a), developed into mature embryos with a visible scutellum within 7 days of culture (Fig. 3b). Around day 10, shoots emerged from the developed embryos (Fig. 3c) and subsequently elongated into plantlets by approximately day 21 (Fig. 3d). Regenerated plantlets were then transferred to 1/2 MS medium for rooting and subsequently acclimatized to soil.

**Table 1:** Effect of callus type on plant regeneration in *Echinochloa phyllopogon*

Callus type	No. of calli	No. of regenerated calli (%)	No. of regenerated plants	No. of regenerated plants/callus
Type 1	63	58 ± 0 (92.1) ^a^	241 ± 16.1 ^a^	4.2 ± 0.3 ^a^
Type 2	63	59 ± 1.7 (94.2) ^a^	248 ± 17.9 ^a^	4.2 ± 0.2 ^a^
Type 3	63	12 ± 0.5 (19.6) ^b^	23 ± 2.6 ^b^	1.9 ± 0.1 ^b^

Values are means ± SD (*n* = 3). Means followed by different letters differ significantly (One-way ANOVA followed by Tukey’s HSD test, *p* < 0.05)

### Transient GUS expression in different callus types

Transient GUS expression after particle bombardment differed significantly among callus types (Table 2). The total number of GUS spots per dish (25 calli) was 1809 and 1825 for Type 1 and Type 2 calli, respectively, both of which were significantly higher than that of Type 3 calli (22 spots). Similarly, the number of GUS spots per callus was approximately 70 for Types 1 and 2, whereas only around 2 spots for Type 3 were observed, indicating markedly higher transformation potential for both Types 1 and 2. Figure 2 show the transient GUS expression in the different calli types. Strong GUS expression was detected in Types 1 and 2 calli, especially in densely packed somatic embryo clusters, whereas little or no staining was observed in Type 3 calli. Both Type 1 and Type 2 calli showed high performance in plant regeneration and transient GUS expression. However, Type 2 calli exhibited superior proliferation and were therefore selected as the target tissue for further transformation experiments.

**Table 2:** Effect of callus type on transient GUS expression in *Echinochloa phyllopogon*

Callus type	No. of bombarded calli	No. of GUS spots	No. of callli expressing GUS (%)	No. of GUS spots/callus
Type 1	25	1809 ± 42.0 ^a^	25 ± 0 (100) ^a^	72 ± 1.7 ^a^
Type 2	25	1825 ± 20.1 ^a^	25 ± 0 (100) ^a^	73 ± 0.8 ^a^
Type 3	25	22 ± 7.4 ^b^	9 ± 0 (45) ^b^	2 ± 0.4 ^b^

Values are means ± SD (*n* = 4). Means followed by different letters differ significantly (One-way ANOVA followed by Tukey’s HSD test, *p* < 0.05)

### Selection and recovery of transgenic plants

After particle bombardment and osmotic treatment, Type 2 calli were transferred to DBC medium supplemented with 5 mg L^−1^ bialaphos and subcultured at 14-day intervals. Almost all bombarded calli gradually turned brown under bialaphos selection. However, bialaphos-resistant cells proliferated and formed visible callus clumps within 90 days (Fig. 1d). These newly formed calli exhibited stable GUS expression, confirming successful transformation (Fig. 1g). The transformation efficiency of Type 2 calli with different subculture durations was summarized in Table 3. Among the callus ages tested, 90-day-old calli had the highest number of putative transgenic calli, with a transformation efficiency of 5.0%. The putative transgenic calli were subsequently transferred to regeneration medium containing 5 mg L^−1^ bialaphos, and all putative transgenic calli derived from 90-day-old calli successfully regenerated into plants (Fig. 1e). The regenerated plants were subsequently transferred to 1/2 MS medium for rooting (Fig. 1f) and after 14–21 days, acclimatized to soil in the growth chamber.

**Table 3:** Effect of embryogenic callus culture duration on transformation efficiency and plant regeneration in *Echinochloa phyllopogon*

Culture duration (days)	No. of bombarded calli	No of GUS-positive calli (%)	No. of regenerated calli (%)
90	240	12 (5.0)	12 (100)
300	240	2 (0.8)	1 (50)
630	120	2 (1.6)	0

### Stable integration and expression of the transgene

**Fig. 4:**
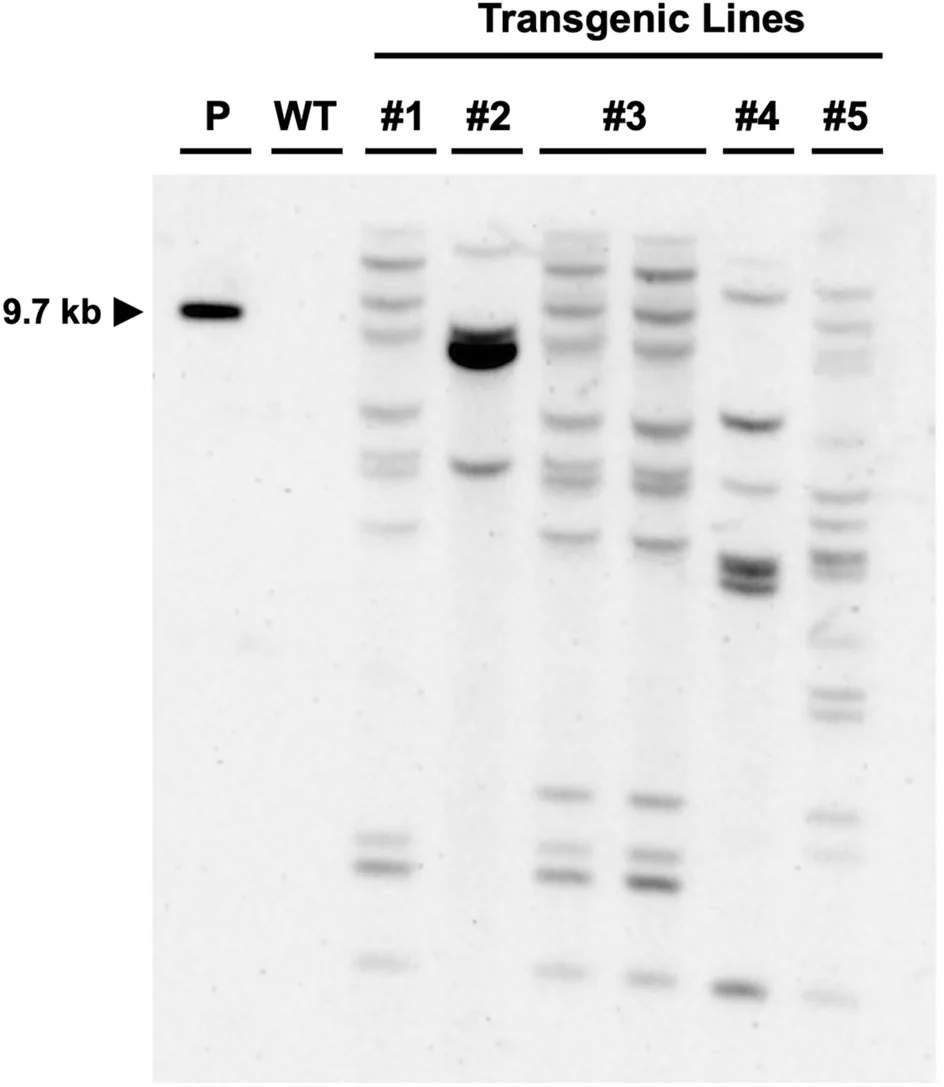
Southern blot analysis of transgenic *Echinochloa phyllopogon* plants. Genomic DNA (4 µg), isolated from leaves of a wild-type plant (WT) and five transgenic lines, was digested with *SacI* and hybridized a DIG-dUTP labeled GUS gene probe. P is positive control of 5 pg *SacI*-digested pAHC25 plasmid DNA. The arrow indicates the expected band (9.7 kb) after Sac I digestion and southern hybridization

Southern blot analysis was performed using the *GUS* gene as a probe to detect the presence of the introduced gene in five independent transgenic lines. All transgenic lines showed hybridization signals corresponding to the introduced gene, with copy numbers between 5 to 12 (Fig. 4). Line #3 consisted of plants regenerated from the same transformed callus and therefore exhibited identical banding patterns.

Stable transgene expression was confirmed by histochemical GUS staining of leaves from acclimatized transgenic plants, which showed strong blue coloration (Fig. 1h). Additionally, herbicide resistance assays were conducted using T_1_ progeny to evaluate the expression of the herbicide-resistant phenotype. Self-pollinated seeds of line #2 were sown and plants at the four-leaf stage were treated with Basta at the recommended concentration (× 1) and a ten-fold higher concentration (× 10). Fifteen days after treatment, all WT plants had died, whereas the transgenic plants of line #2 continued to grow normally at the recommended concentration and survived even at the higher concentration (Fig. 1i), demonstrating transmission of the herbicide-resistant phenotype to the T_1_ generation.

## Discussion

Functional characterization of candidate genes is essential for elucidating the molecular mechanisms of multiple herbicide resistance in *E. phyllopogon*. In particular, verification of genes involved in herbicide metabolism and detoxification requires a stable genetic transformation system. However, no stable transformation protocol has previously been reported for this important C4 weed species. Therefore, establishing an efficient and reproducible transformation system is a critical prerequisite for advancing our understanding of herbicide resistance mechanisms in *E. phyllopogon*.

In this study, we established a genetic transformation system for *E. phyllopogon* using particle bombardment, in which callus type was a critical factor affecting both plant regeneration and transformation efficiency. Type 1 and Type 2 calli, characterized by embryogenic structures, showed high regeneration ability and transient GUS expression, whereas Type 3 calli exhibited poor performance (Fig. 2, Tables 1, 2). This transformation system was based on our previously established protocols for C4 warm-season forage grasses (Muguerza et al. 2022), including *Paspalum notatum* (Gondo et al. 2005), *Brachiaria ruziziensis* (Ishigaki et al. 2012), *Pennisetum purpureum* (Gondo et al. 2017) and *Zoysia matrella* (Ng et al. 2023). In these systems, the quality of embryogenic callus is particularly important, and the use of highly regenerative embryogenic calli with dense pre-embryogenic clusters on the surface is a key factor for successful transformation. Similar observations have been reported in major cereals such as rice (Hiei et al. 1994) and maize (Ishida et al. 1996). In these studies, actively proliferating embryogenic calli were found to significantly enhance transformation efficiency. These findings are consistent with our results, highlighting the importance of high-quality, actively proliferating embryogenic callus for efficient transformation.

Calli cultured for 90 days exhibited the highest transformation efficiency (5.0%), and all transgenic calli successfully regenerated into plants (Table 3). In contrast, calli subcultured for 300 and 630 days showed reduced transformation efficiency and most transgenic calli could not be regenerated. These results suggest that long-term culture of callus not only leads to changes in embryonic structure and a decline in regenerative capacity, but also could increase the risk of somaclonal variation, including the occurrence of albinism (Cho et al. 1998; Lambé et al. 1998; Gondo et al. 2005). Recent genome-wide studies have further demonstrated that tissue culture can substantially increase the accumulation of somatic mutations, raising concerns regarding genomic integrity during clonal propagation (Matthew et al 2026). Therefore, minimizing the duration of callus culture may be important not only for maintaining regeneration capacity but also for reducing the risk of tissue culture-induced genetic variation.

Stable integration and expression of the introduced gene were confirmed by Southern blot analysis and histochemical GUS staining. All transgenic lines contained multiple copies of the transgene (5–12 copies), which is consistent with previous reports indicating that particle bombardment frequently results in relatively high transgene copy numbers (Pawlowski and Somers 1996; Kohli et al. 1998). Nevertheless, consistent GUS expression in acclimatized plants indicated stable gene expression. In addition, herbicide resistance assays performed in the T_1_ generation demonstrated that transgenic plants exhibited strong resistance to Basta, even at concentrations ten times higher than the recommended level, whereas wild-type plants were completely susceptible. These results demonstrate stable integration and functional expression of the introduced gene in the T_0_ generation and indicate that the herbicide-resistant phenotype was transmitted to the T_1_ generation.

Although *Agrobacterium*-mediated transformation has been successfully applied to several grass species (Muguerza et al. 2022), its efficiency is highly species- and genotype-dependent. In our preliminary experiments using *Agrobacterium*-mediated transformation with *E*. *phyllopogon*, neither *Agrobacterium* infection nor transient GUS expression was detected. Therefore, particle bombardment was selected for establishing a genetic transformation system in this species. To our knowledge, this is the first successful report of transgenic *E*. *phyllopogon* plants.

In conclusion, we successfully established the first genetic transformation system for *E. phyllopogon*. The results demonstrated that both callus type and culture duration are critical factors influencing plant regeneration and transformation efficiency, with highly embryogenic calli and relatively short-term cultured calli (approximately 90 days) showing the best performance. The established system enabled stable integrationand expression of the introduced gene, with the herbicide-resistant phenotype observed in the T_1_ generation. This transformation platform will facilitate functional analysis of candidate genes involved in herbicide metabolic detoxification and contribute to elucidating the molecular mechanisms underlying multiple herbicide resistance of NTSR in this species.

## Data Availability

No datasets were generated or analysed during the current study.
